# Computation of the Spatial Distribution of Charge-Carrier Density in Disordered Media

**DOI:** 10.3390/e26050356

**Published:** 2024-04-24

**Authors:** Alexey V. Nenashev, Florian Gebhard, Klaus Meerholz, Sergei D. Baranovskii

**Affiliations:** 1 Faculty of Physics, Philipps-Universität Marburg, 35032 Marburg, Germany; florian.gebhard@physik.uni-marburg.de; 2 Department für Chemie, Universität zu Köln, Greinstraße 4-6, 50939 Köln, Germany

**Keywords:** disordered materials, electron states in random potential

## Abstract

The space- and temperature-dependent electron distribution n(r,T) determines optoelectronic properties of disordered semiconductors. It is a challenging task to get access to n(r,T) in random potentials, while avoiding the time-consuming numerical solution of the Schrödinger equation. We present several numerical techniques targeted to fulfill this task. For a degenerate system with Fermi statistics, a numerical approach based on a matrix inversion and one based on a system of linear equations are developed. For a non-degenerate system with Boltzmann statistics, a numerical technique based on a universal low-pass filter and one based on random wave functions are introduced. The high accuracy of the approximate calculations are checked by comparison with the exact quantum-mechanical solutions.

## 1. Introduction

Disordered materials, such as amorphous organic and inorganic semiconductors and semiconductor alloys, play an important role in modern optoelectronics for computing, communications, photovoltaics, sensing, and light emission [1,2,3,4,5,6,7,8]. The spatial and energy disorder creates a random potential, that decisively affects electron states. Among other effects, the disorder potential causes spatial localization of electrons in the low-energy range. The knowledge of the space- and temperature-dependent electron distribution n(r,T), particularly in localized states, created by random potentials, is required to understand charge transport and light absorption/emission in disordered semiconductors. The distribution n(r,T) is most straightforwardly obtained by solving the Schrödinger equation in the presence of disorder potential. However, this procedure is extremely demanding with respect to computation facilities. It is hardly affordable for realistically large chemically complex systems. Therefore, it is highly desirable to develop theoretical tools to get access to n(r,T) without solving the Schrödinger equation.

One of the currently mostly used theoretical tools to reveal the individual features of localized states in a random potential is the so-called localization-landscape theory (LLT) [9,10,11,12]. In the LLT, the random potential is converted into some effective potential, drastically simplifying the calculations. However, recent studies [13,14] revealed substantial problems of the LLT. For instance, the effective potential in the LLT lacks the temperature dependence, which is necessary to describe n(r,T) appropriately. Moreover, the LLT has been proven equivalent to the Lorentzian filter applied to a random potential [13]. Such a choice of the filter function is rather unfortunate. The Lorentzian filter yields a significantly larger number of localized states in a random potential than the number of such states obtained via the exact solution of the Schrödinger equation [13]. Therefore, more-developed computational techniques are desirable.

Here, we develop two numerical techniques to reveal n(r,T) in disordered systems under degenerate conditions controlled by Fermi statistics, avoiding the time-consuming numerical solution of the Schrödinger equation. One of the techniques is based on converting the Hamiltonian into a matrix, which, being subjected to several multiplications with itself, succeeded by inverting the outcome, yields the distribution n(r,T). The other technique replaces the operation of matrix inversion by solving a system of linear equations controlled by the matrix generated from the Hamiltonian.

We also describe two recently developed computational techniques for calculations of n(r,T) in non-degenerate systems controlled by Boltzmann statistics [13,14]. One algorithm is based on applying a temperature-dependent universal low-pass filter (ULF) to the random potential V(r). This yields a temperature-dependent effective potential, W(r,T), that enables a quasiclassical calculation of particle density n(r,T). The ULF algorithm employs fast Fourier transformation for calculating the effective potential *W*, enabling the analysis of very large systems.

The other algorithm is based on a recursive application of the Hamiltonian to multiple sets of random wave functions (RWFs) for a specific realization of the random potential V(r). Following the repeated application of the thermal operator, the temperature-dependent electron density is determined by averaging the outcomes over different RWF sets. This procedure offers several advantages over the widely used LLT. Unlike the LLT, which relies on an adjustable parameter that can only be determined through comparison with the exact solution [13,14], the RWF scheme does not involve adjustable parameters, and it works across all temperatures. Additionally, the accuracy of the RWF approach in computing n(r,T) can systematically be improved, whereas the accuracy of the LLT is inherently limited.

## 2. Calculation of n(r,T) in a Degenerate System Controlled by Fermi Statistics

To be definite, we consider a disorder potential characterized by Gaussian statistics (‘white noise’), i.e., the potential obeys ${\langle V\left(r\;\right)\rangle }_{R}=0$ with the auto-correlation function [15]

(1) $${\langle V\left(r\;\right)V\left(r^{\prime }\right)\rangle }_{R}=S\delta \left(r-r^{\prime }\right)\;,$$

where ${\langle \ldots \rangle }_{R}$ indicates the average over many realizations R of the random potential and *S* is the strength of the interaction. The quantity *S* yields natural definitions for the characteristic length scale and for the characteristic energy scale in the form

(2) $$\ell_{0}={\left(\frac{\hbar^{4}}{m^{2}S}\right)}^{1/(4-d)}\;,$$

(3) $$T_{0}=\frac{1}{k_{B}}{\left(\frac{m^{d}S^{2}}{\hbar^{2d}}\right)}^{1/(4-d)}\;,$$

where *d* is the space dimensionality and $k_{B}$ is the Boltzmann constant.

We consider a collection of non-interacting electrons in some external potential in the thermodynamic equilibrium characterized by the temperature *T* and the Fermi energy $\varepsilon_{f}$. The goal is to develop an effective numerical method to calculate the electron density (concentration) n(r,T) as a function of coordinates r in a degenerate system. The electron density can be defined as

(4) $$n\left(r,T\right)=2\sum_{a}{\left|\psi_{a}\left(r\right)\right|}^{2}f\left(\varepsilon_{a}\right).$$

In this equation, summation index *a* labels the eigenstates, i.e., solutions of the Schrödonger equation $\hat{H}\psi_{a}=\varepsilon_{a}\psi_{a}$, where $\psi_{a}\left(r\right)$ and $\varepsilon_{a}$ are the wavefunction and the energy of the eigenstate, f(ε) is the Fermi function,

(5) $$f\left(\varepsilon \right)=\frac{1}{\exp[\left(\varepsilon -\varepsilon_{f}\right)/k_{B}T]+1}\;,$$

and factor 2 in front of the sum in Equation (4) accounts for the two possible spin orientations.

We assume that the system under study is discretized with a finite-difference (or tight-binding) method. The tight-binding method is widely used for modeling of various transport phenomena in the presence of disorder, ranging from Anderson localization to disordered topological insulators. As a few examples, let us mention studies of one-dimensional disordered chains [16,17,18], tight-binding models of topological insulators in amorphous systems [19], tight-binding models for lead halide perovskites [20], and nucleic acid sequences [21]. A wavefunction ψ(r) is, therefore, represented as a collection of probability amplitudes $\psi_{1},\ldots ,\psi_{L}$ at *L* points (grid nodes) evenly distributed in space. The Hamiltonian $\hat{H}$ is a matrix of size L×L. Equation (4) for n(r,T), in this discrete setting, attains the form

(6) $$n_{i}=\frac{2}{\Delta V}\sum_{a=1}^{L}{\left|\psi_{a,i}\right|}^{2}f\left(\varepsilon_{a}\right),$$

where $n_{i}$ is the electron density at the grid node i∈{1,…,L}, $\psi_{a,i}$ is the value of the eigenfunction $\psi_{a}$ at node *i*; $\varepsilon_{a}$ is the electron energy that corresponds to this eigenfunction; and ΔV is the spatial volume per one grid node (in the one-dimensional case, ΔV is simply the distance between nodes).

Equation (6) represents a standard way to calculate numerically the electron density in degenerate systems. However, this way requires the solution of the eigenvalue problem, which takes a large amount of computational resources for the large size *L* of the Hamiltonian matrix. Below, we suggest two methods that allow one to speed up the calculation of n(r,T). In Method 1 (see Section 2.1), the numerical solution of the eigenvalue problem is replaced by the matrix inversion. The latter numerical task is much faster than the solution of the eigenvalue problem in the case of a *sparse matrix*, in which almost all entries are equal to zero. In Method 2 (see Section 2.2), we employ a numerical solution of a system of linear equations that is even faster than the matrix inversion. The performances of Methods 1 and 2, as compared to the standard method based on the eigenvalue problem, are tested in Section 2.3 on the example of a one-dimensional disordered system with one occupied band.

The idea of Methods 1 and 2 is based on a simple observation that the shape of the function

(7) $$y\left(x\right)=\frac{1}{x^{N}+1}\;,$$

where a number N is large, resembles the shape of the Fermi function in the vicinity of point x=1. Other approaches for approximating the Fermi function are also possible [22,23,24,25]. The essence of Methods 1 and 2 is the replacement of *x* with an appropriate linear function of the Hamiltonian. The detailed justification is provided in Appendix A.

It is implicitly assumed in this section that the Fermi energy is inside the energy gap far from the band edges, as the model Hamiltonians exclude two-particle interaction. In a situation, when the two-particle interaction is unavoidable, it can be included into the model via the exchange-correlation potential of the density functional theory. The latter issue is out of scope in our study.

### 2.1. Method 1: Matrix Inversion

The inputs of Method 1 are the Hamiltonian $\hat{H}$ (a matrix L×L), the Fermi energy $\varepsilon_{f}$, and two additional parameters, a “reference energy” $\varepsilon_{0}$ and the number of iterations *N*. The three latter parameters are related to temperature *T* via the equality

(8) $$k_{B}T=\frac{|\varepsilon_{f}-\varepsilon_{0}|}{2^{N}}\;.$$

The details for the choice of these parameters are given in Appendix B.

In Method 1, one first composes the matrix ${\hat{A}}_{0}$ from the Hamiltonian,

(9) $${\hat{A}}_{0}=\frac{\hat{H}-\varepsilon_{0}\hat{I}}{\varepsilon_{f}-\varepsilon_{0}}\;,$$

where $\hat{I}$ is the L×L unit matrix. This matrix ${\hat{A}}_{0}$ is then squared *N* times

(10) $${\hat{A}}_{p}={\left({\hat{A}}_{p-1}\right)}^{2},\;p=1,2,\ldots ,N.$$

Afterwards, the unit matrix is added to matrix ${\hat{A}}_{N}$ and the sum is inverted to yield a new matrix

(11) $$\hat{B}={\left({\hat{A}}_{N}+\hat{I}\right)}^{-1}.$$

Finally, the electron density is obtained from the diagonal elements $B_{ii}$ of the matrix $\hat{B}$:

(12) $$n_{i}=\frac{2}{\Delta V}\;B_{ii}\;\mathrm{if}\;\varepsilon_{0}<\varepsilon_{f}\;,$$

(13) $$n_{i}=\frac{2}{\Delta V}\;\left(1-B_{ii}\right)\;\mathrm{if}\;\varepsilon_{0}>\varepsilon_{f}\;.$$

### 2.2. Method 2: Solving a System of Linear Equation

**Figure 1:**
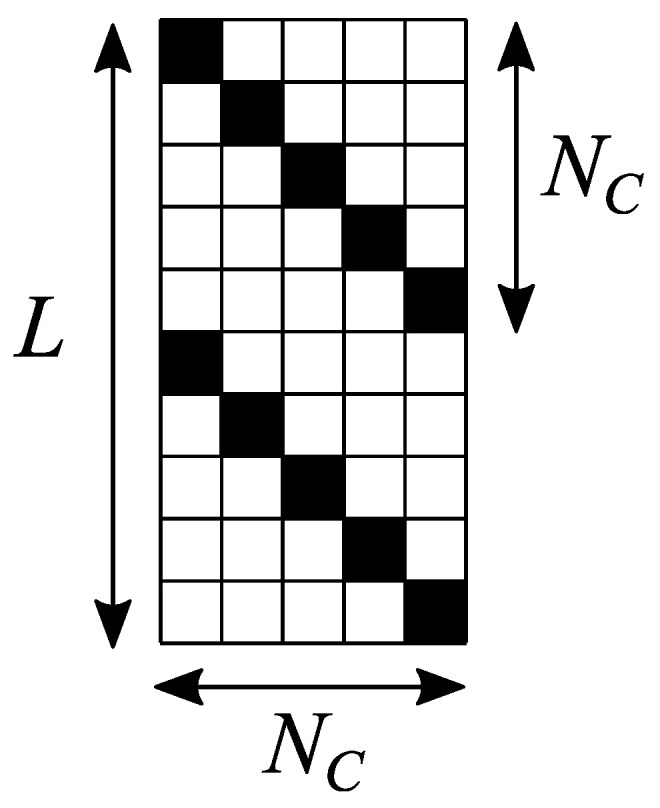
Sketch of matrix $\hat{U}$ for the one-dimensional case. White and black squares represent zeros and unities, respectively.

Similarly to Method 1, the input parameters are $\varepsilon_{f}$, $\varepsilon_{0}$ and *N*, which are related to temperature *T* by Equation (8). In addition, Method 2 requires one extra parameter $N_{C}$. The choice of $N_{C}$ is discussed in Appendix B. For a given $N_{C}$, a matrix $\hat{U}$ of size $L\times N_{C}$ is to be composed, that fulfills the following conditions (see Figure 1 for the shape of this matrix in the one-dimensional case):

- each entry of matrix $\hat{U}$ is equal to either 0 or 1;
- in each row of matrix $\hat{U}$, exactly one entry is equal to 1;
- in each column of matrix $\hat{U}$, the nodes with nonzero entries are placed spatially as far from each other as possible. For example, in the one-dimensional case, the unities in each column are separated by $\left(N_{C}-1\right)$ zeros, as illustrated in Figure 1.

The matrix ${\hat{A}}_{N}$ is calculated, as done in Method 1, see Equations (9) and (10). However, in contrast to Method 1, no matrix inversion is necessary. Instead, a system of linear equations

(14) $$\left({\hat{A}}_{N}+\hat{I}\right)\hat{X}=\hat{U}$$

is to be solved with respect to the yet unknown matrix $\hat{X}$ of size $L\times N_{C}$. This is a computationally easier task than the matrix inversion used in Method 1.

Finally, the electron density is calculated as

(15) $$n_{i}=\frac{2}{\Delta V}\;\sum_{a=1}^{N_{C}}X_{ia}U_{ia}\;\mathrm{if}\;\varepsilon_{0}<\varepsilon_{f}\;,$$

(16) $$n_{i}=\frac{2}{\Delta V}\;\left(1-\sum_{a=1}^{N_{C}}X_{ia}U_{ia}\right)\;\mathrm{if}\;\varepsilon_{0}>\varepsilon_{f}\;.$$

### 2.3. A Numerical Example: One-Dimensional Disordered System with a Single Occupied Band

**Figure 2:**
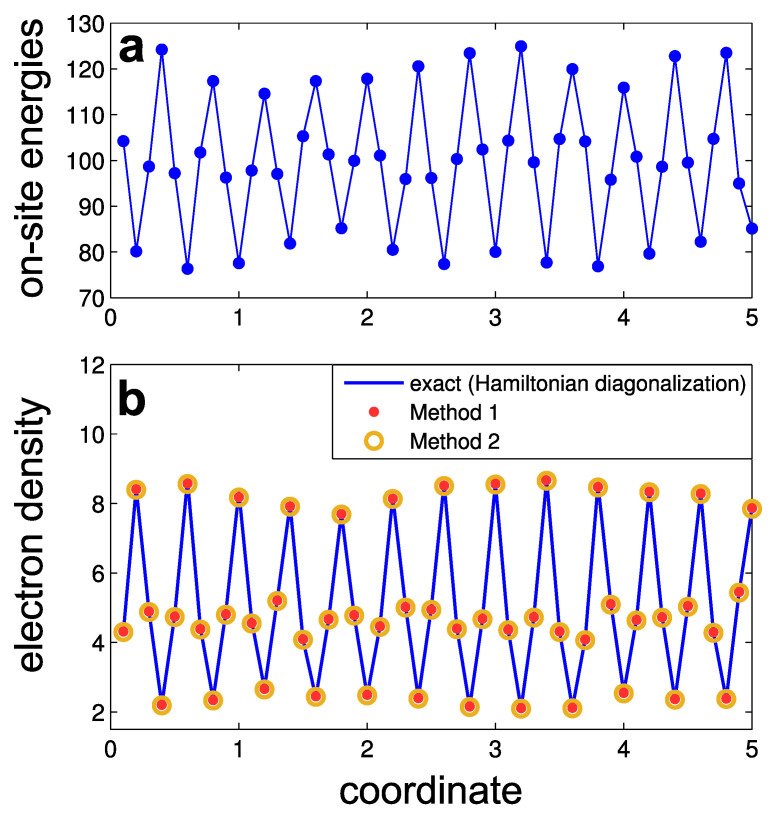
Numerical example of one-dimensional tight-binding model: (**a**) on-site energies $H_{jj}$ at different nodes; (**b**) electron density in the lowest energy band calculated by three methods: exact diagonalization of the Hamiltonian (Equation (18), blue lines), Method 1 (red dots), and Method 2 (orange circles).

Let us compare the performances of different methods in calculating n(r,T) on a simple one-dimensional tight-binding model with energy bands and disorder. We consider a linear chain of *L* lattice nodes at the distance a=0.1 from each other. The distances and energies are measured in the units determined by Equations (2) and (3). In these units, the hopping integrals between the neighboring nodes $H_{i,i+1}$ and $H_{i+1,i}$ are equal to $-1/(2a^{2})=-50$. The on-site energies $H_{jj}$ are chosen to be

(17) $$H_{jj}=V_{0}+V_{1}\cos\left(\pi j/2\right)+V_{2}\xi_{j}\;,$$

where $\xi_{j}$ are the random numbers uniformly distributed in the range $-1<\xi_{j}<1$. All the other matrix elements of the Hamiltonian $\hat{H}$ are equal to zero. Periodic boundary conditions apply. The constant term $V_{0}=100$ makes the lower boundary of the energy spectrum $\varepsilon_{\min}$ close to zero, and the higher boundary is $\varepsilon_{\max}\approx 200$. The amplitude of the periodic variations of the potential is $V_{1}=20$. The amplitude of the random-noise potential $V_{2}=\sqrt{30}$ is chosen to set the ’disorder strength’ *S* equal to unity. This relation can be understood as follows. In a discretized 1D model, the Dirac delta function $\delta (x_{i}-x_{j})$ turns into (1/a)δ(i,j), where δ(i,j) is a Kronecker delta, and *a* is the distance between sites. Hence, Equation (1), reformulated with the discrete variables, has the form ${\langle V_{i}\;V_{j}\rangle }_{R}=\left(S/a\right)\delta \left(i,j\right)$. Inserting potentials $V_{2}\xi_{i}$, $V_{2}\xi_{j}$ yields $V_{2}^{2}{\langle \xi_{i}\;\xi_{j}\;\rangle }_{R}=\left(S/a\right)\delta \left(i,j\right)$. Since ${\langle \xi_{i}\;\xi_{j}\;\rangle }_{R}=\left(1/3\right)\delta \left(i,j\right)$, one obtains $V_{2}=\sqrt{3S/a}=\sqrt{30}$ for the assumed values S=1 and a=0.1. As an example, we show one realization of the on-site energies in Figure 2a.

As a consequence of the periodic potential (the second term in the right-hand side of Equation (17)), the electron energy spectrum consists of the four energy bands separated from each other by band gaps. We consider the situation when the lowest energy band is completely occupied by electrons, and three other bands are empty (quarter filling). The electron density in such a case is expressed as

(18) $$n_{i}=\frac{2}{\Delta V}\sum_{a\in \mathrm{OB}}{\left|\psi_{a,i}\right|}^{2},$$

where summation is performed over the eigenstates of the occupied band.

The wave functions $\psi_{a}$, that enter Equation (18), can be obtained by diagonalizing the Hamiltonian. An example of the calculated electron density distribution is shown in Figure 2b by blue lines.

Also we show in Figure 2b the approximated electron density obtained by Method 1 (red dots) and by Method 2 (orange circles). The parameters for these methods are: $\varepsilon_{f}=28.5$ (the middle of the lowest band gap), $\varepsilon_{0}=10$, N=3, and $N_{C}=30$. One can see that both Methods provide quite accurate results for the electron density.

**Figure 3:**
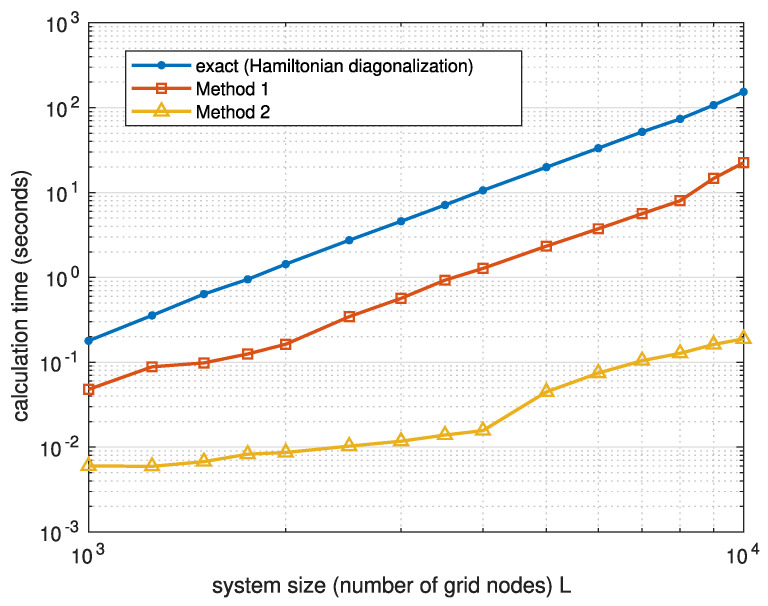
The average calculation time for the electron density in the one-dimensional tight-binding model shown in Figure 2 as a function of the number of nodes *L*. Different curves correspond to different numerical methods.

In Figure 3, we compare the time required by three ways of calculating the electron density—the exact method based on the Hamiltonian diagonalization, Method 1, and Method 2—on a desktop PC with MATLAB (version R2023b, by MathWorks) used for the matrix manipulations. One can see that Method 1, which employs matrix inversion, is approximately one order of magnitude faster than the usual method of Hamiltonian diagonalization. Method 2 provides additional increase in speed by more than an order of magnitude.

Note that Method 1 can be further improved by using advanced techniques to calculate the diagonal part of the inverted matrix [26,27,28]. For the sake of simplicity, we use here the standard MATLAB functions in Method 1. Even in such a non-optimized setting, this method demonstrates a substantial speedup in comparison with matrix diagonalization.

So far, we considered here degenerate systems with Fermi statistics. In Section 3, we address a complementary case of a non-degenerate system with Boltzmann statistics, following two recent studies [13,14]. While in those studies, hundreds of equations were used to treat the non-degenerate case, we demonstrate in this paper that the essence of the theory for Boltzmann statistics can be introduced using only several equations.

## 3. Calculation of n(r,T) in a Non-Degenerate System Controlled by Boltzmann Statistics

### 3.1. Low-Pass Filter (LF) Approach

#### 3.1.1. Motivation

**Figure 4:**
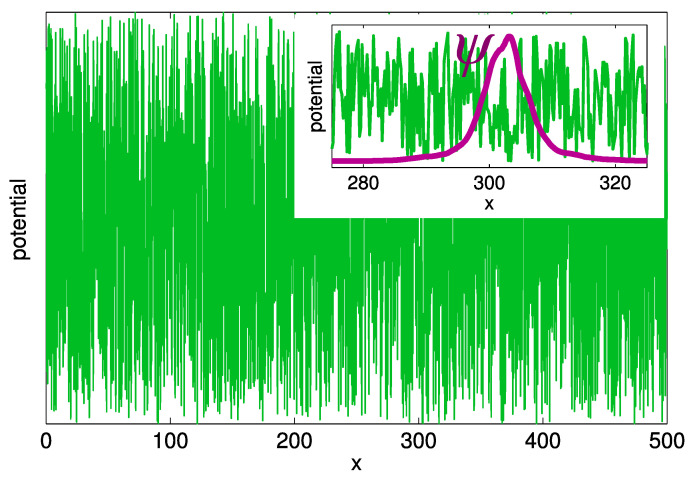
Realization of the white-noise disorder potential on a one-dimensional strip. Insert: the wave function ψ(x) of the state with the lowest energy in the region 275≤x≤325. The coordinate *x* is dimensionless in the units given by Equation (2).

Already in the 1960s, Halperin and Lax recognized that electrons in a random potential cannot follow very short-range potential fluctuations [15]. This effect is illustrated in Figure 4, where the random white-noise potential V(x) in one dimension is depicted by the green solid line. The detailed shape of V(x) in the region 275≤x≤325 (in the dimensionless units given by Equation (2)) is compared with the shape of the wave function ψ for the lowest energy state in this spatial region. Apparently, the characteristic width $\ell_{wf}$ of the wave function, even for low-energy localized states, is substantially larger than the spatial scale of the fluctuations of the disorder potential V(r). The latter scale in semiconductor alloys is of the order of the lattice constant a≈0.5 nm. The strong inequality $\ell_{wf}\gg a$ suggests that electrons in localized states are affected only by the mean disorder potential, averaged over the space scale $\ell_{wf}$. It is, therefore, not necessary to solve exactly the Schrödinger equation with the real disorder potential in order to get access to the individual features of electron states. Instead, one can apply to the disorder potential V(r) a low-pass filter [15] (LF) that smooths the spatial fluctuations of V(r).

Halperin and Lax suggested the square of the wave function as the filter function [15]. The width of the filter function, $\ell \approx \ell_{wf}$ was adjusted dependent on the energy, ε, of the localized state, $\ell \propto {\left|\varepsilon \right|}^{-1/2}$, where ε is counted from the band edge in the absence of disorder. A variational approach was used to determine the shape of the low-energy density of states in a random potential [15]. Baranovskii and Efros [29] addressed the same problem by a slightly different variational approach and confirmed the result of Halperin and Lax.

Neither of the two groups considered, however, the individual features of localized states, being focused solely on the structure of the density of states in the low-energy region [15,29]. Our aim here is, in contrast, to calculate the spatial distribution of electron density, n(r,T), in a given disorder potential V(r). We start below with the definition of the low-pass filter and then formulate the algorithm to calculate n(r,T). Afterwards, we will formulate an even more accurate technique for calculating n(r,T) based on the random-wave-function approach.

#### 3.1.2. Definition of a Low-Pass Filter (LF)

In one dimension, the low-pass filter (LF) is determined by the operation

(19) $$W\left(x\right)=\int dx^{\prime }\Gamma \left(x-x^{\prime }\right)V\left(x^{\prime }\right)\;,$$

where the filter function Γ should contain the appropriate length scale *ℓ*. This operation converts the real disorder potential V(x) into the smooth effective potential W(x). For instance, one can try a Lorentzian function $\Gamma^{L}$,

(20) $$\Gamma^{L}\left(x\right)=\frac{e^{-\left|x\right|/\ell_{L}}}{2\ell_{L}}\;,$$

because using as LF a Lorentzian function with $\ell_{L}=0.27\;\ell_{0}$ has recently been proven [13] to be equivalent to the popular LLT approach [9,10,11,12].

**Figure 5:**
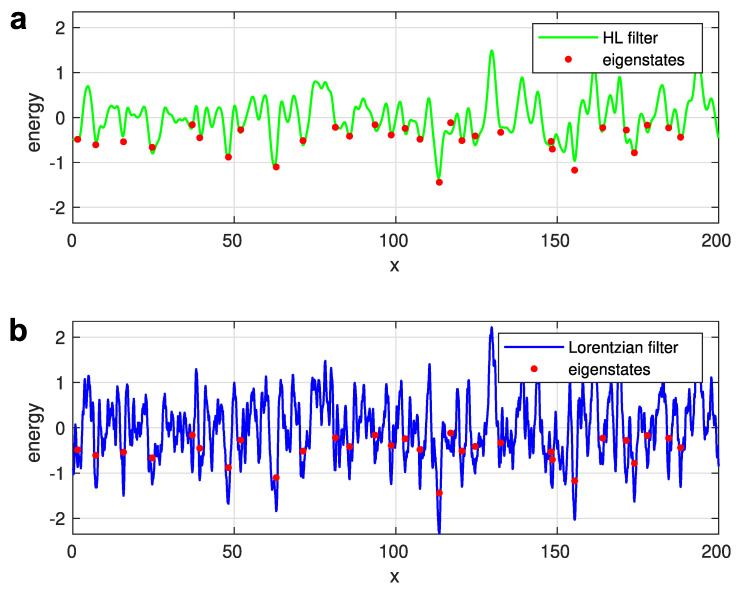
(**a**) Effective potential for a Halperin-Lax low-pass filter. (**b**) Effective potential for a Lorentzian low-pass filter. Reprinted with permission from [13]. Copyright (2023) by the American Physical Society.

Halperin and Lax [15] suggested instead to use for LF the square of the wave function $\Gamma \left(x\right)=\psi^{2}\left(x\right)$. The shape of the wave functions ψ(x) for the low-energy states was determined by the optimal-fluctuation-approach that yields the filtering function

(21) $$\Gamma^{\mathrm{HL}}\left(x\right)=\frac{1}{2\ell_{\mathrm{HL}}}\frac{1}{\cosh^{2}\left(x/\ell_{\mathrm{HL}}\right)}\;,$$

where the characteristic length $\ell_{\mathrm{HL}}$ should depend on the state energy [15,29]. Remarkably, it appears that a universal, energy-independent value for $\ell_{\mathrm{HL}}$, can be introduced [13], $\ell_{\mathrm{HL}}=0.76\;\ell_{0}$ as evidenced in Figure 5a, where the effective potential yielded by the filtering function given by Equation (21), is compared with the positions and energies of the eigenstates. The eigenstates for the given realization of disorder potential V(x) were obtained via a straightforward solution of the Schrödinger equation. In Figure 5, the 30 eigenstates, with the lowest energies are depicted by red points. The excellent agreement between the local minima of the effective potential (shown by the solid green line) and the positions and energies of the exactly calculated eigenstates justifies the filter function given by Equation (21).

In Figure 5b, such a comparison is illustrated for the case of a Lorentzian filter, determined by Equation (20) with $\ell_{L}=0.27\;\ell_{0}$ chosen to mimic the LLT result [13]. Evidently, the choice of a Lorentzian filter function is not satisfying. The number of the local minima in the effective potential W(x) (shown by the solid blue line) is significantly larger than the true number of the exactly calculated eigenstates. This happens because the Lorentzian function given by Equation (20) is not smooth at x=0. The cusp at x=0 filters too many extrema from the real disorder potential V(x), preventing the identification of true localized electron states by searching the minima of the effective potential W(x). The filter function suggested by Halperin and Lax [15] does not possess such a deficiency.

Not only the energies and spatial positions of localized states in disorder potential V(r), discussed above, are of interest for the theory. In fact, the key quantity for the optoelectronic properties of disordered semiconductors is the space- and temperature-dependent electron distribution n(r,T). Below we extend the LF approach to calculate n(r,T). For that purpose, we introduce the temperature *T* into the filter function and, concomitantly, into the definition of the effective potential W(r,T) that yields n(r,T).

#### 3.1.3. Universal Filter Function to Determine n(r,T)

The *T*-dependent spatial distribution of electron density n(r,T) is related to the quasi-classical effective potential W(r,T) as

(22) $$n\left(r,T\right)=N_{c}\exp\left[\frac{\mu -W(r,T)}{k_{B}T}\right]\;,$$

where $N_{c}$ is the effective density of states in the conduction band and μ is the chemical potential. This equation serves as the definition of the quasi-classical effective potential W(r,T). The effective potential is smooth in comparison to the initial disorder potential V(r) because W(r,T) is derived from the electron density n(r,T), which has the spatial scale of the electron wave functions, i.e., is broader than the scale of the short-range fluctuations of V(r). Let us, therefore, obtain W(r,T) by subjecting V(r) to the action of a universal low-pass filter (ULF).

The key question is how to find out the appropriate *T*-dependent filter function Γ(r,T) that can be used to extract the shape of the effective potential W(r,T) for a given realization of the white-noise potential V(r). One can represent this filter function as a functional derivative

$$\Gamma \left(r-r^{\prime },T\right)=\frac{\delta W(r,T)}{\delta V(r^{\prime })}\;.$$

This derivative can be calculated analytically via the first-order perturbation theory in the approximation of vanishing function V(r), i.e., for the free electron gas. This perturbation approach yields the following shape of the filter function’s Fourier image [14]:

(23) $$\hat{\Gamma }\left(k\right)=\frac{\sqrt{\pi }}{\lambda k}\;e^{-\lambda^{2}k^{2}/4}\;\mathrm{erfi}\left(\lambda k/2\right)\;,$$

where erfi is the imaginary error function, and $\lambda =\hbar /\sqrt{2mk_{B}T}$.

**Figure 6:**
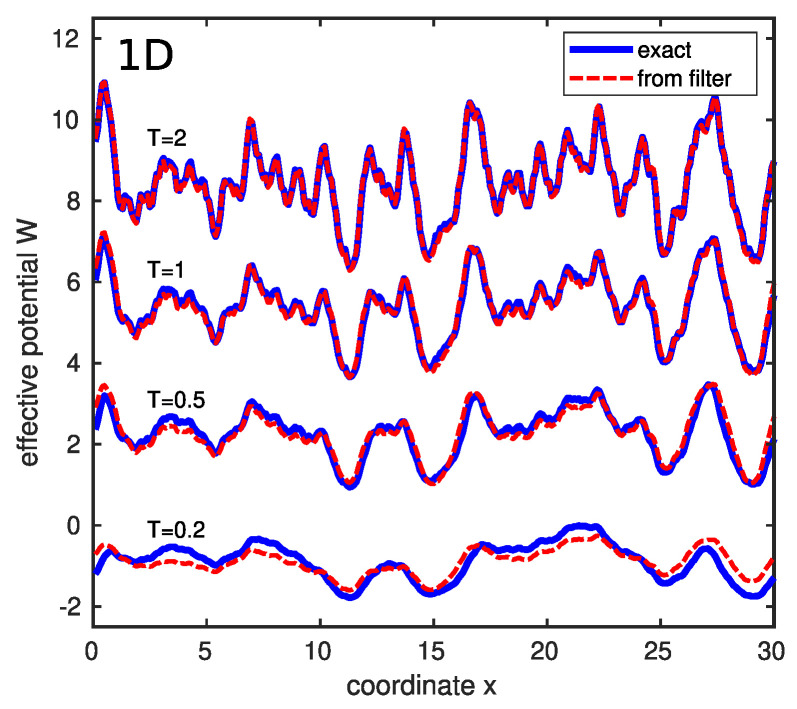
Comparison between the exact effective potential (solid blue lines) and the filtered potential (dashed red lines) for a one-dimensional sample with white-noise potential. Reprinted with permission from [14]. Copyright (2023) by the American Physical Society.

**Figure 7:**
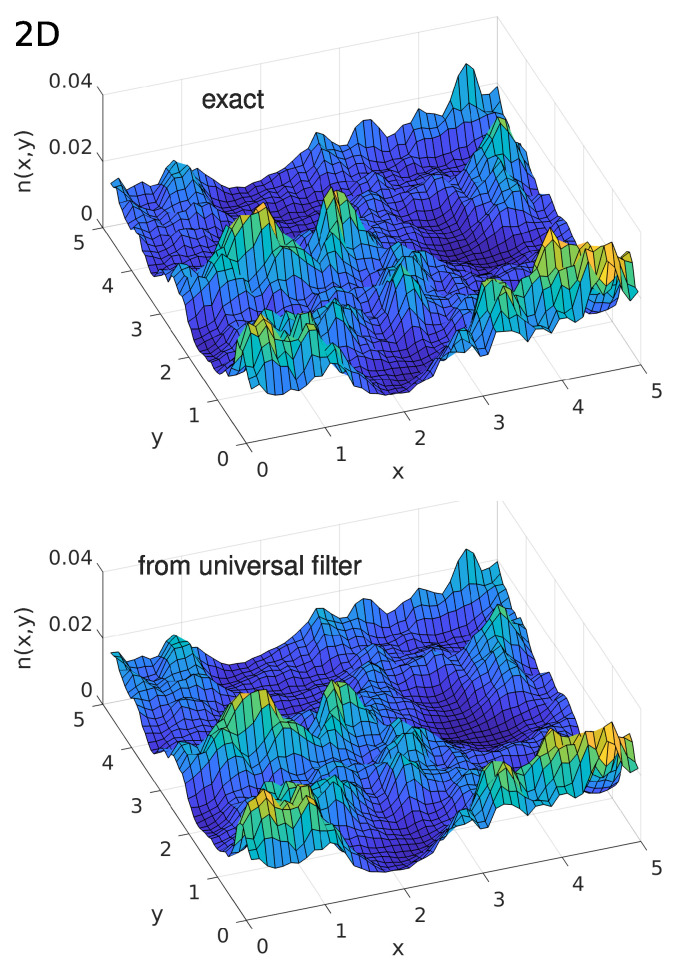
Comparison between the exact electron density n(x,y,T) (**upper part**) and that obtained by using the universal filter function (**lower part**) in a two-dimensional white-noise potential. Reprinted with permission from [14]. Copyright (2023) by the American Physical Society.

In order to reveal the electron density distribution n(r,T) for a given realization of the white-noise potential V(r), one should first calculate the Fourier image $\hat{V}\left(k\right)$ of V(r) using a fast-Fourier-transform (FFT). This function $\hat{V}\left(k\right)$ should be then multiplied by $\hat{\Gamma }\left(|k|\right)$, which is given by Equation (23). The inverse Fourier transform of the product $\hat{V}\left(k\right)\hat{\Gamma }\left(|k|\right)$ by the FFT yields the effective potential W(r,T) because the inverse Fourier transform converts a product into a convolution [14]. Inserting W(r,T) into Equation (22) gives the electron density n(r,T). In Figure 6 and Figure 7, we compare the results for W(r,T) of the above procedure with the effective potentials obtained via Equation (22) from the electron density n(r,T) calculated using the exact solution of Schrödinger equation in one and two dimensions, respectively. Coordinate *x* and temperature *T* in the figures are measured in the units given by Equations (2) and (3).

The data in Figure 6 and Figure 7 demonstrate the high accuracy of the approach based on the *T*-dependent low-pass filter. Notably, the FFT operation used in this approach does not need a considerable computation time, in contrast to the exact calculations on the basis of Schrödinger equation.

Below, we introduce an even more accurate technique to calculate electron density n(r,T) while avoiding solving the Schrödinger equation. This technique is based on the random-wave-functions approach.

### 3.2. Random Wave Functions (RWF) Approach to Calculate n(r,T)

#### 3.2.1. Background

The idea of the RWF approach resembles the one suggested recently by Lu and Steinerberger [30] to search for the low-lying eigenfunctions of various linear operators. An iterative application of the operator leads to the increasing contributions of the low-energy regions to the state vector [30]. A similar approach has been suggested by Krajewski and Parrinello [31] for the calculation of the thermodynamic potential.

Let us consider the action of the operator $\hat{h}=\exp\left[-\hat{H}/\left(2k_{B}T\right)\right]$ on an arbitrarily chosen wave function. The goal is to model the equilibrium distribution of electrons, which is described by the Boltzmann statistics in the nondegenerate case considered here. In Boltzmann statistics, states with energy ε contribute to the distribution of electrons with the probability proportional to $\exp[-\varepsilon /\left(k_{B}T\right)]$. The wave function is the probability amplitude, which explains the factor 1/2 in the operator $\hat{h}$. The wave function is always a linear combination of eigenfunctions that correspond to different energies. The action of the operator $\hat{h}$ suppresses the contributions of high-energy eigenfunctions in favor of the contributions of low-energy eigenfunctions. By the application of the operator $\hat{h}$ to a collection of the random wave functions, the average contributions of eigenfunctions corresponding to different energies approach their distribution in thermal equilibrium. The averaging here is performed over the set of the random wave functions. Physically, this procedure corresponds to the averaging of the electron density n(r,T). The question arises on how to numerically subject a wave function to the action of the operator $\hat{h}=\exp\left[-\hat{H}/\left(2k_{B}T\right)\right]$. This can be done by recursively applying the Hamiltonian $\hat{H}$ to the wave function:

(24) $$\hat{h}=e^{-\hat{H}/2k_{B}T}\approx {\left(1-\alpha \hat{H}\right)}^{M}\;,$$

with a natural number [14] $M\approx 1/(2\alpha k_{B}T)$ and a small parameter α. A simple analysis justifies the choice [14] $\alpha =1.5/\epsilon_{\max}$, where $\epsilon_{\max}$ is the estimate of the upper boundary of the energy distribution. In the case of a regular grid with the lattice constant *a*, $\epsilon_{\max}≃\hbar^{2}/\left(ma^{2}\right)$. Below, we describe how to realize this idea technically.

#### 3.2.2. The RWF Algorithm

Let us consider the RWF algorithm on a spatial lattice with the volume ΔV per lattice site. The value of the random wave function ψ on each lattice site is chosen independently as a random number extracted from a normal distribution with the average value zero and variance 1/ΔV. The following transformation of the wave function ψ,

(25) $$\psi \to \psi -\alpha \hat{H}\psi \;$$

is applied *M* times. Then, an estimate of the reduced electron density ${\tilde{n}}_{R}\left(r,T\right)$ is

(26) $${\tilde{n}}_{R}\left(r,T\right)=2\;{\left|\psi \left(r\right)\right|}^{2}\;.$$

The calculation of ${\tilde{n}}_{R}\left(r,T\right)$ is carried out for a large number $N_{R}$ of realizations R of the random wave function ψ(r). Then, the electron density n(r,T) is the arithmetic mean of the functions ${\tilde{n}}_{R}\left(r\right)$ obtained for different realizations R, multiplied by a chemical-potential-related factor $e^{\mu /(k_{B}T)}$,

(27) $$n\left(r,T\right)=e^{\mu /(k_{B}T)}{\langle {\tilde{n}}_{R}\left(r,T\right)\rangle }_{R}\;.$$

The larger the number of realizations $N_{R}$ is, the more accurate the calculated electron density n(r,T) will be.

**Figure 8:**
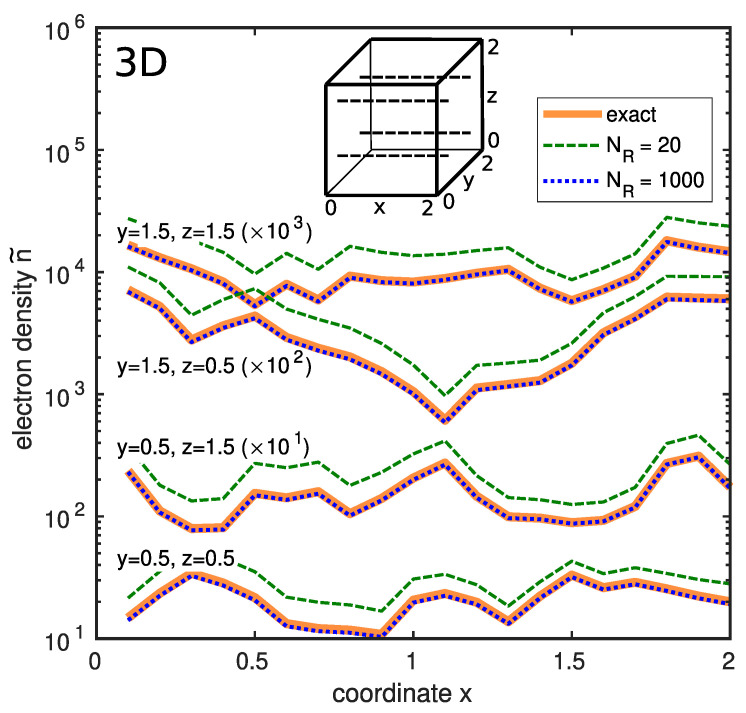
Reduced electron density $\tilde{n}\left(x,y,z,T\right)$ in a three-dimensional sample with white-noise potential. Compared are the exact density (solid orange lines) with that obtained by the RWF algorithm (dashed green lines for $N_{R}=20$ and dotted blue lines for $N_{R}=1000$). Profiles along the *x*-axis with different values of coordinates *y* and *z* are shown (see inset). Sample size is 2×2×2 dimensionless units, the discretization grid parameter is a=0.1, the temperature is $T=T_{0}$. Periodic boundary conditions apply. For clarity, profiles are multiplied by different coefficients, as indicated in the plot. Reprinted with permission from [14]. Copyright (2023) by the American Physical Society.

In Figure 8, the reduced electron densities obtained via the RWF algorithm are compared with the exact one calculated using the solution of the Schrödinger equation for three dimensions. Evidently, the RWF algorithm with 1000 iterations accurately yields the electron density.

## 4. Conclusions

In this work, we introduce four theoretical tools to get access to the space- and temperature-dependent electron density n(r,T) in disordered media with a random potential V(r), avoiding the time-consuming numerical solution of the Schrödinger equation.

For the case of degenerate conditions controlled by Fermi statistics, the Hamiltonian is converted into a matrix, which, being subjected to several multiplications with itself, succeeded by inverting the outcome, yields the distribution n(r,T). The other possible technique for the case of Fermi statistics replaces the operation of matrix inversion by solving a system of linear equations controlled by the matrix generated from the Hamiltonian.

For non-degenerate conditions with Boltzmann statistics, the universal low-pass filter (ULF) approach and the random-wave-function (RWF) algorithm are suggested for approximate calculations of n(r,T). Both methods require far less computational resources than the complete solution of the Schrödinger equation.

The ULF approach employs the temperature-dependent effective potential W(r,T). This technique is based on the Fast Fourier Transformation, which does not impose any demands on computational resources, such as processor time and memory. Therefore, it can be applied to mesoscopically large three-dimensional disordered systems. Being superior to the widely used approximate methods, the RWF is computationally more costly than the ULF approach when mesoscopically large three-dimensional systems at low temperatures are addressed. However, the accuracy of calculations based on the RWF algorithm can be unlimitedly improved by increasing the number of the RWF realizations.

## Figures and Tables

**Figure A1:**
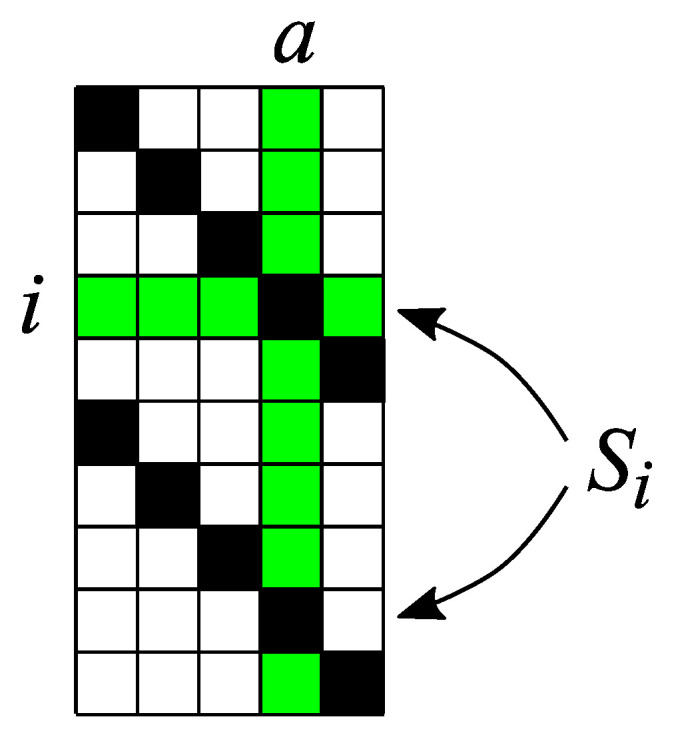
Illustration of set $S_{i}$ for a given row index *i* of matrix $\hat{U}$. White and black squares represent zeros and ones, respectively. Row *i* and column *a* of the matrix are highlighted in green.

**Figure A2:**
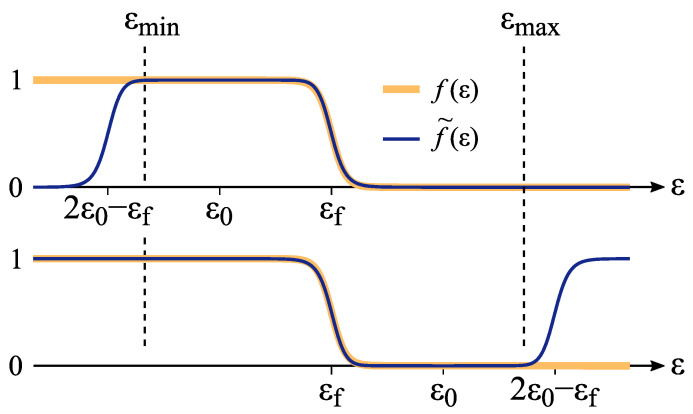
Comparison of the Fermi function f(ε) and function $\tilde{f}\left(\varepsilon \right)$. Above: the case of $\varepsilon_{0}<\varepsilon_{f}$, below: the case of $\varepsilon_{0}>\varepsilon_{f}$.

## Data Availability

The data presented in this study are available on request from the corresponding author.

## Abbreviations

LLT, localization landscape theory; ULF, universal low-pass filter; RWF, random wave functions; LF, low-pass filter; FFT, fast Fourier transform.

## Appendix A. Why Do Methods 1 and 2 Work

To understand why Method 1 works, let us apply to the Hamiltonian $\hat{H}$ a unitary transformation that diagonalizes it. This transformation also diagonalizes all the matrices that appear in Method 1. In accordance with Equations (9)–(11), the diagonal elements of matrices $\hat{H}$, ${\hat{A}}_{0}$, ${\hat{A}}_{N}$ and $\hat{B}$ are

(A1) $$\begin{array}{cc} H_{aa} & =\varepsilon_{a}\;, \end{array}$$

(A2) $$\begin{array}{cc} A_{0,aa} & =\frac{\varepsilon_{a}-\varepsilon_{0}}{\varepsilon_{f}-\varepsilon_{0}}\;, \end{array}$$

(A3) $$\begin{array}{cc} A_{N,aa} & ={\left(\frac{\varepsilon_{a}-\varepsilon_{0}}{\varepsilon_{f}-\varepsilon_{0}}\right)}^{2^{N}}\;, \end{array}$$

(A4) $$\begin{array}{cc} B_{aa} & =\frac{1}{{\left(\frac{\varepsilon_{a}-\varepsilon_{0}}{\varepsilon_{f}-\varepsilon_{0}}\right)}^{2^{N}}+1}\;. \end{array}$$

Let us consider the values of $\varepsilon_{a}$ that are close to the Fermi energy $\varepsilon_{f}$. These values obey the relation

(A5) $${\left(\frac{\varepsilon_{a}-\varepsilon_{0}}{\varepsilon_{f}-\varepsilon_{0}}\right)}^{2^{N}}={\left(\frac{\varepsilon_{a}-\varepsilon_{f}}{\varepsilon_{f}-\varepsilon_{0}}+1\right)}^{2^{N}}\approx \exp\left(2^{N}\frac{\varepsilon_{a}-\varepsilon_{f}}{\varepsilon_{f}-\varepsilon_{0}}\right).$$

Taking Equation (8) into account, one can rewrite this estimate as

(A6) $${\left(\frac{\varepsilon_{a}-\varepsilon_{0}}{\varepsilon_{f}-\varepsilon_{0}}\right)}^{2^{N}}\approx \exp\left(\pm \;\frac{\varepsilon_{a}-\varepsilon_{f}}{k_{B}T}\right),$$

where sign “+” is chosen if $\varepsilon_{0}<\varepsilon_{f}$, and sign “−” is chosen in the opposite case. Substitution of Equation (A6) into Equation (A4), with further comparison to expression (5) for the Fermi function f(ε), provides the relations

(A7) $$B_{aa}\approx f\left(\varepsilon_{a}\right)\;\mathrm{if}\;\varepsilon_{0}<\varepsilon_{f}\;,$$

(A8) $$1-B_{aa}\approx f\left(\varepsilon_{a}\right)\;\mathrm{if}\;\varepsilon_{0}>\varepsilon_{f}\;,$$

which arise after the unitary transformation that diagonalizes the Hamiltonian. Upon this transformation, Equation (A7) acquires the form

(A9) $$B_{ij}\approx \sum_{a=1}^{L}f\left(\varepsilon_{a}\right)\psi_{a,i}\psi_{a,j}^{*}\;\mathrm{if}\;\varepsilon_{0}<\varepsilon_{f}\;,$$

yielding for the diagonal matrix elements of the matrix $\hat{B}$ the relation

(A10) $$B_{ii}\approx \sum_{a=1}^{L}f\left(\varepsilon_{a}\right){\left|\psi_{a,i}\right|}^{2}\;\mathrm{if}\;\varepsilon_{0}<\varepsilon_{f}\;,$$

or, due to Equation (6),

(A11) $$B_{ii}\approx \frac{\Delta V}{2}\;n_{i}\;\mathrm{if}\;\varepsilon_{0}<\varepsilon_{f}\;.$$

This justifies Equation (12) of Method 1.

Similarly, from Equation (A8) one can get after applying the unitary transformation, the relation

(A12) $$1-B_{ii}\approx \frac{\Delta V}{2}\;n_{i}\;\mathrm{if}\;\varepsilon_{0}>\varepsilon_{f}\;,$$

justifying Equation (13) of Method 1.

Let us now turn to Method 2. For simplicity, we restrict consideration to the case $\varepsilon_{0}<\varepsilon_{f}$. The case of the opposite inequality can be treated in a similar way. According to Equations (11) and (14), matrix $\hat{X}$ in Equation (14) can be expressed as $\hat{X}=\hat{B}\hat{U}$. Hence, the sum in the right-hand side of Equation (15) can be rewritten as

(A13) $$\sum_{a=1}^{N_{C}}X_{ia}U_{ia}=\sum_{a=1}^{N_{C}}\sum_{j=1}^{L}B_{ij}U_{ja}U_{ia}\;.$$

According to construction of the matrix $\hat{U}$ (see Section 2.2), there is only one value of *a* for a given *i*, such that $U_{ia}=1$. For this value of *a*, there are only a few values of *j* such that $U_{ja}=1$, as illustrated in Figure A1. Let us denote the set of these values of *j* as $S_{i}$. One can then rewrite Equation (A13) as

(A14)
$$\sum_{a=1}^{N_{C}}X_{ia}U_{ia}=\sum_{j\in S_{i}}B_{ij}\;.$$

Note that $i\in S_{i}$ since $U_{ia}=1$. Therefore, the sum in the right-hand side of Equation (A14) contains the term $B_{ii}$. Notably, all other terms $B_{ij}$ in this sum with j≠i are negligible at sufficiently large $N_{C}$. Indeed, one can see from Equation (A9) that the matrix $\hat{B}$ is close to the projector $\hat{P}$ to the set of eigenstates with energies below the Fermi energy. Particularly, in the situation when the filled states are separated by a band gap from the empty ones, the matrix elements $P_{ij}$ of this projector decrease with increasing distance between nodes *i* and *j*, and become negligible at some distance. Hence, matrix elements $B_{ij}$ in the right-hand side of Equation (A14) become negligible if nodes *i* and *j* are far away from each other, i.e., if i≠j. The only non-negligible term is $B_{ii}$, and therefore,

(A15) $$\sum_{a=1}^{N_{C}}X_{ia}U_{ia}\approx B_{ii}\;.$$

Substituting $B_{ii}$ from Equation (A11), one arrives at Equation (15) of Method 2.

Similarly, the combination of Equations (A12) and (A15) justifies Equation (16) of Method 2 in the case $\varepsilon_{0}>\varepsilon_{f}$.

## Appendix B. Choice of Parameters *ε*_0_, *N* and *N*_*C*_

There are two restrictions on the choice of parameters $\varepsilon_{0}$ and *N* and, hence, on the temperature value *T*, for which Methods 1 and 2 are valid. First, the approximate Equalities (A7) and (A8) must hold over the whole range of electron energies. Second, the matrix $\left({\hat{A}}_{N}+\hat{I}\right)$ must not be ill-conditioned.

To consider the first restriction, let us define the function $\tilde{f}\left(\varepsilon \right)$ as

(A16) $$\tilde{f}\left(\varepsilon \right)=\frac{1}{{\left(\frac{\varepsilon -\varepsilon_{0}}{\varepsilon_{f}-\varepsilon_{0}}\right)}^{2^{N}}+1}\;\mathrm{if}\;\varepsilon_{0}<\varepsilon_{f}\;,$$

(A17) $$\tilde{f}\left(\varepsilon \right)=1-\frac{1}{{\left(\frac{\varepsilon -\varepsilon_{0}}{\varepsilon_{f}-\varepsilon_{0}}\right)}^{2^{N}}+1}\;\mathrm{if}\;\varepsilon_{0}>\varepsilon_{f}\;.$$

Methods 1 and 2 are based on the fact that this function is close to the Fermi function f(ε) in the vicinity of the Fermi energy $\varepsilon_{f}$ (see Equations (A4), (A7) and (A8)). Let us consider the behavior of the function $\tilde{f}\left(\varepsilon \right)$ in a broader range of energies ε.

As an example, Figure A2 shows $\tilde{f}\left(\varepsilon \right)$ along with the Fermi function f(ε) for N=4 and two choices of the “reference energy” $\varepsilon_{0}$: $\varepsilon_{0}<\varepsilon_{f}$ (upper panel) and $\varepsilon_{0}>\varepsilon_{f}$ (lower panel). In both cases, there is a discrepancy between the functions f(ε) and $\tilde{f}\left(\varepsilon \right)$ below the energy $2\varepsilon_{0}-\varepsilon_{f}$ in the first case, and above the energy $2\varepsilon_{0}-\varepsilon_{f}$ in the second case. Electron energy levels must not fall into these areas of discrepancy, otherwise the contributions of these levels into the electron density would not be accounted for correctly in Methods 1 and 2. Therefore, in the case of $\varepsilon_{0}<\varepsilon_{f}$, the lowest electron energy $\varepsilon_{\min}$ must be larger than $2\varepsilon_{0}-\varepsilon_{f}$. Similarly, in the case of $\varepsilon_{0}>\varepsilon_{f}$, the highest electron energy $\varepsilon_{\max}$ must be smaller than $2\varepsilon_{0}-\varepsilon_{f}$. These conditions can be rewritten as restrictions to the “reference energy” $\varepsilon_{0}$:

(A18) $$\varepsilon_{0}<\frac{\varepsilon_{f}+\varepsilon_{\min}}{2}\;\mathrm{or}\;\varepsilon_{0}>\frac{\varepsilon_{f}+\varepsilon_{\max}}{2}\;.$$

Let us now consider the second restriction. The inversion of the matrix $\left({\hat{A}}_{N}+\hat{I}\right)$ in Method 1, or solving a system of linear equations expressed by this matrix in Method 2, is possible when this matrix is not ill-conditioned. This means that the condition number, a ratio of the largest and the smallest eigenvalues of the matrix, is less than the maximal value of the order of $10^{15}$ (a number that corresponds to the accuracy of representation of real numbers in the computer memory). The minimal eigenvalue of matrix $\left({\hat{A}}_{N}+\hat{I}\right)$ is close to unity, and corresponds to the energy levels close to $\varepsilon_{0}$. The largest eigenvalue corresponds to the energy level farthest from $\varepsilon_{0}$, i. e., either to $\varepsilon_{\min}$ or to $\varepsilon_{\max}$, and is approximately the largest of the two values ${\left[\left(\varepsilon_{\min}-\varepsilon_{0}\right)/\left(\varepsilon_{f}-\varepsilon_{0}\right)\right]}^{2^{N}}$ and ${\left[\left(\varepsilon_{\max}-\varepsilon_{0}\right)/\left(\varepsilon_{f}-\varepsilon_{0}\right)\right]}^{2^{N}}$. Hence, the parameters $\varepsilon_{0}$ and *N* have to obey the following restrictions:

(A19) $${\left(\frac{\varepsilon_{\min}-\varepsilon_{0}}{\varepsilon_{f}-\varepsilon_{0}}\right)}^{2^{N}}<10^{15}\;\mathrm{and}\;{\left(\frac{\varepsilon_{\max}-\varepsilon_{0}}{\varepsilon_{f}-\varepsilon_{0}}\right)}^{2^{N}}<10^{15}\;.$$

The choice of parameters $\varepsilon_{0}$ and *N* is based on Equations (A18) and (A19). We consider two different options: (i) the temperature *T* is given; (ii) the goal is to obtain the sharpest possible boundary between filled and empty states.

In the first option, one should try the natural numbers in ascending order as values of *N*. For each of such numbers *N*, one should find $\varepsilon_{0}$ according to Equation (8) and check whether conditions (A18) and (A19) are fulfilled. The smallest suitable value of *N* is the best choice. Indeed, the smaller *N* is, the more sparse matrix $\left({\hat{A}}_{N}+\hat{I}\right)$ will be and, consequently, the faster is matrix inversion in Method 1 or solution of linear equations in Method 2 will be.

In the second option, the largest possible *N* is desirable, in order to minimize the temperature *T* according to Equation (8). To achieve the largest *N*, one has to choose the value $\varepsilon_{0}$ that maximizes the denominator $|\varepsilon_{f}-\varepsilon_{0}|$ in Equation (A19):

(A20) $$\varepsilon_{0}\approx \frac{\varepsilon_{f}+\varepsilon_{\min}}{2}\;\mathrm{or}\;\varepsilon_{0}\approx \frac{\varepsilon_{f}+\varepsilon_{\max}}{2}\;,$$

and then one should choose the maximal number *N* that obeys the restrictions (A19),

(A21) $$N\approx \log_{2}\left(\frac{15}{\log_{10}\left|\frac{\varepsilon_{\max}-\varepsilon_{0}}{\varepsilon_{f}-\varepsilon_{0}}\right|}\right)$$

or

(A22) $$N\approx \log_{2}\left(\frac{15}{\log_{10}\left|\frac{\varepsilon_{\min}-\varepsilon_{0}}{\varepsilon_{f}-\varepsilon_{0}}\right|}\right)$$

for the first and the second choice of $\varepsilon_{0}$ in Equation (A20), respectively.

Finally, let us consider the choice of parameter $N_{C}$ in Method 2. The accuracy of Method 2 improves with rising $N_{C}$. However, larger $N_{C}$ cause a longer computation time due to the increase in the size of the matrix $\hat{U}$ in Equation (14). Hence, there is a trade-off between the accuracy and the efficiency. The best value of parameter $N_{C}$ can be estimated as

(A23) $$N_{C}≃{\left(\ell /a\right)}^{d},$$

where *d* is the dimensionality of the space, *a* is the distance between neighboring sites in the lattice, and *ℓ* is a characteristic decay length of the matrix element $B_{ij}$ with increasing distance between sites *i* and *j*.

A question might arise on whether the minimum $N_{C}$ value increases with increasing disorder that forms tails in the density of states at the edges of the respective subbands. Such an effect is unlikely, because the tail states are strongly localized. Therefore, their contributions into projector $\hat{P}\left(r\;,r^{\prime }\right)$ would not enlarge its decay length *ℓ*.

The minimum value of $N_{C}$ is not directly related to the dimension of sub-bands. Whereas the sub-band dimension is proportional to the sample volume, the value of $N_{C}$ is determined by the characteristic decay length *ℓ* of the projector $\hat{P}\left(r\;,r^{\prime }\right)$ onto the subspace of occupied states with increasing distance $|r-r^{\prime }|$. The relation between the minimum value of $N_{C}$ and the length *ℓ* is given by Equation (A23). The idea behind using the restricted value of $N_{C}$ for an arbitrarily large sample is that, due to the decay of projector $\hat{P}\left(r\;,r^{\prime }\right)$ with the distance $|r-r^{\prime }|$, the matrix $\hat{B}$ defined by Equation (11) consists mostly of zeros. The number of non-negligible elements per each row of matrix $\hat{B}$ is the minimal value of $N_{C}$, which provides the estimate of $N_{C}$ given in Equation (A23). The information stored in matrix $\hat{B}$ of size L×L (where *L* is the dimension of the sample) can therefore be “packed” into a much smaller matrix $\hat{X}=\hat{B}\hat{U}$ of size $L\times N_{C}$. Hence, in order to obtain the electron density that is stored in the diagonal elements $B_{ii}$ of matrix $\hat{B}$, it is sufficient to calculate the matrix $\hat{X}$. This idea is rationalized in Appendix A; see the passage between Equations (A14) and (A15).
